# Renal Protective Role of Xiexin Decoction with Multiple Active Ingredients Involves Inhibition of Inflammation through Downregulation of the Nuclear Factor-*κ*B Pathway in Diabetic Rats

**DOI:** 10.1155/2013/715671

**Published:** 2013-07-02

**Authors:** Jia-sheng Wu, Rong Shi, Jie Zhong, Xiong Lu, Bing-liang Ma, Tian-ming Wang, Bin Zan, Yue-ming Ma, Neng-neng Cheng, Fu-rong Qiu

**Affiliations:** ^1^Department of Pharmacology, Shanghai University of Traditional Chinese Medicine, Shanghai 201203, China; ^2^Experiment Center for Science and Technology, Shanghai University of Traditional Chinese Medicine, Shanghai 201203, China; ^3^Department of Pharmacology, School of Pharmacy, Fudan University, Shanghai 201203, China; ^4^Laboratory of Clinical Pharmacokinetics, Shanghai Shuguang Hospital, Shanghai 201203, China

## Abstract

In Chinese medicine, Xiexin decoction (XXD) has been used for the clinical treatment of diabetes for at least 1700 years. The present study was conducted to investigate the effective ingredients of XXD and their molecular mechanisms of antidiabetic nephropathy in rats. Rats with diabetes induced by high-fat diet and streptozotocin were treated with XXD extract for 12 weeks. XXD significantly improved the glucolipid metabolism disorder, attenuated albuminuria and renal pathological changes, reduced renal advanced glycation end-products, inhibited receptor for advanced glycation end-product and inflammation factors expression, suppressed renal nuclear factor-**κ**B pathway activity, and downregulated renal transforming growth factor-**β**1. The concentrations of multiple components in plasma from XXD were determined by liquid chromatography and tandem mass spectrometry. Pharmacokinetic/pharmacodynamic analysis using partial least square regression revealed that 8 ingredients of XXD were responsible for renal protective effects via actions on multiple molecular targets. Our study suggests that the renal protective role of XXD with multiple effective ingredients involves inhibition of inflammation through downregulation of the nuclear factor-**κ**B pathway, reducing renal advanced glycation end-products and receptor for advanced glycation end-product in diabetic rats.

## 1. Introduction

The number of diabetic patients is increasing rapidly worldwide [1]. Diabetic nephropathy (DN) is one of the main microvascular complications of diabetes, and also the main cause of end-stage renal disease [2]. The pathogenesis of DN is complicated. Hyperglycaemia induces renal injury through multiple pathways, including the polyol pathway [3], the protein kinase C pathway [4], generation of advanced glycation end-products (AGEs) [5], oxidative stress [6], and inflammation [7, 8]. Previous studies have shown that the development of DN is a slow process. Clinical measures currently used to control blood glucose and blood pressure and to inhibit the renin-angiotensin system can delay this process [9–11]. However, the number of patients whose disease has progressed from diabetes mellitus to end-stage renal failure continues to increase, even if these measures have been adopted [12]. Therefore, new drugs must be researched and developed to prevent the occurrence and development of DN more effectively. 

Traditional Chinese medicine (TCM) has been used to treat diabetes mellitus for several thousand years [13]. Recent studies have shown that Chinese herbal compounds significantly promote recovery in experimental diabetes and its complications [14–17]. These findings imply that TCM could be useful clinically for the treatment of diabetes mellitus and its complications. The development of new drugs based on classical TCM compounds is an important approach for TCM translational medicine research. Xiexin decoction (XXD) is a classic Chinese herbal preparation containing Radix et Rhizoma Rhei (*Rheum palmatum L.*), Rhizoma Coptidis (*Coptis chinensis* Franch), and Radix Scutellaria (*Scutellaria baicalensis* Georgi) in the ratio of 2 : 1 : 1 (w/w). It has been used for the treatment of diabetic mellitus (called xiaoke disease in TCM) since the Tong Dynasty (6th century C.E.) [18, 19]. Our previous studies showed that XXD had beneficial effects on early-stage DN [20]. However, the molecular mechanism of action of XXD is not yet clear, thereby limiting further research and development. Studies showed that XXD had obvious anti-inflammatory effects [21–23]. The relationship between the anti-DN effect and the anti-inflammatory effects of XXD is not clear. This research aimed to elucidate the molecular mechanism underlying XXD's anti-DN activity, with a focus on its anti-inflammatory effects.

**Figure 1:**
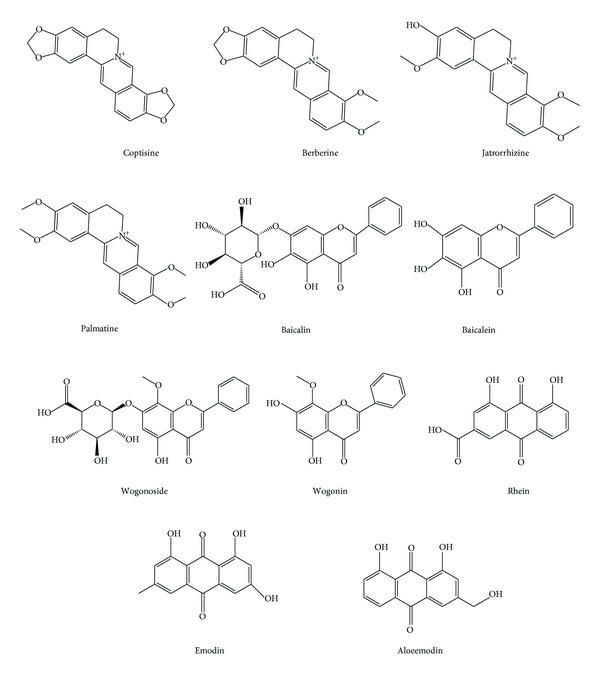
Chemical structures of Xiexin decoction ingredients.

Chinese herbal compounds with multiple ingredients always act on many targets simultaneously to generate a range of actions that manifest as a comprehensive overall effect. Our previous studies [24] showed that 11 ingredients were measurable in rat plasma after oral administration of XXD, including coptisine, jatrorrhizine, berberine, palmatine, baicalin, baicalein, wogonoside, wogonin, rhein, emodin, and aloeemodin (Figure 1). However, the effective ingredients for the anti-DN effect of XXD *in vivo* are currently unclear. Moreover, the relationship between the effective ingredients and their molecular mechanisms is also not clear, thereby limiting further research and development of XXD. A combined pharmacokinetics/pharmacodynamics (PK/PD) approach can be used to identify the effective ingredients in TCM. However, the traditional PK/PD model is not suitable because the time-effect relationship is not always clear after TCM administration; repeated administrations can cause obvious effects; and multiple ingredients simultaneously act on multiple targets to cause different effects [25]. After the administration of TCM, the relationships between the range of ingredients present and their effects on multiple targets *in vivo* are very complicated. The partial least squares (PLS) regression method provides a linear regression model for the analysis of the relationships between multiple dependent, and multiple independent, variables. This method, which has some advantages over traditional regression analysis, can be used to analyse complicated relationships between 2 sets of multiple variables [26]. This approach has been successfully applied in quantitative structure-activity relationship analysis [27], quantitative structure-PK relationship analysis [28], metabolomic analysis [29], and analysis of the relationships between genes and disease [30]. The present study aimed to perform a PK/PD model analysis using the PLS regression method to investigate the relationship between the ingredients of XXD and its anti-DN effect and to explore viable research methods for analysis of the active ingredients of TCM.

## 2. Materials and Methods

### 2.1. Antibodies

For western blot analysis, polyclonal anti-bodies of transforming growth factor-*β*1 (TGF-*β*1), nuclear factor-*κ*Bp65 (NF-*κ*Bp65), inhibitor of nuclear factor *κ*B kinase subunit *α* (IKK*α*), inhibitor of nuclear factor *κ*B subunit *α* (I*κ*B*α*), phospho-NF-*κ*Bp65, and phospho-I*κ*B*α* were obtained from Cell Signaling Technology, USA. Polyclonal antibodies of receptor for AGE (RAGE), intercellular adhesion molecule-1 (ICAM-1), monocyte chemotactic protein-1 (MCP-1), and *β*-actin were obtained from Santa Cruz Biotechnology, USA.

### 2.2. Preparation and High-Pressure Liquid Chromatography (HPLC) Analysis of XXD

Herbs present in XXD, including Radix et Rhizoma Rhei, Rhizoma Coptidis, and Radix Scutellaria, were purchased from the Shanghai Kang Qiao herbal pieces Co. (Shanghai, China). Authentication of these herbs was performed by Professor Zhi-Li Zhao, Department of Botany, Shanghai University of TCM, China. XXD was prepared as previously described [24]. Simultaneous quantification of 11 typical ingredients of this extract (Table 1) was performed using HPLC methods [31, 32].

### 2.3. Animals and Diabetic Model

Male Sprague-Dawley rats (90−100 g) were purchased from the Shanghai Slac Laboratory Animal Co. (Shanghai, China). The rats were housed in an air-conditioned room at 22–24°C under a 12-h dark/light cycle and were given food and water at libitum. All animal experiments were conducted in accordance with the institutional guidelines for the care and use of laboratory animals at Shanghai University of TCM. After 1-week adaptation, animals were divided into a normal control (NC) group fed a standard diet and a high-fat group received a high-fat diet. After 4 weeks, rats on the high-fat diet were treated with streptozotocin (40 mg/kg, intraperitoneal injection). All diabetic rats with fasting blood glucose (FBG) levels above 16.7 mmol/L were then randomly divided into 5 groups: diabetic model control (DM); XXD extract 1.25 g/kg (DM + XXDL); XXD extract 2.5 g/kg (DM + XXDH); losartan 10 mg/kg (DM + Losartan); and Metformin 100 mg/kg (DM + metformin). In the clinical practice of TCM, XXD is usually prescribed at a daily dose of 46 g of herbal materials (amount to 12 g extract) for diabetic patient [18]. When this human dose was converted into an animal dose (a person of 60 kg, and a conversion factor of 6.25 between human and rat), it was equivalent to the low dose (1.25 g/kg extract) used in this study. NC and DM were treated with vehicle (normal saline) in a matched volume. All the rats were administered the drugs via intragastric gavage (ig) once a day, for 12 weeks.

### 2.4. Pharmacokinetics Study

The rats treated with XXD for 12 weeks were fasted with free access to water for 12 h before the PK experiments. Blood samples were collected before dosing and at 0, 0.25, 0.5, 1, 2, 4, 6, 12, and 24 h following administration. A validated liquid chromatography-tandem mass spectrometry (LC-MS/MS) method [24] was applied to simultaneously determine the concentration of 11 ingredients (coptisine, jatrorrhizine, berberine, palmatine, baicalin, baicalein, wogonoside, wogonin, rhein, emodin, and aloeemodin) in blood plasma [24]. The plasma concentration-time data were analysed by noncompartmental methods with the WinNonLin software package (Pharsight Corporation, Mountain View, CA, USA) to determine PK parameters.

### 2.5. Urinary Albumin Excretion, Metabolic Parameters, and Renal Function Analysis

At 4, 8, and 12 weeks, 24 h urine of rat was collected for measurements of 24 h urinary albumin excretion (UAE) by radioimmunoassay (Atom High Technology, Beijing, China). At 12 weeks, FBG and area under the blood glucose response curve (GAUC) were measured by glucose oxidase method. HbA_1c_ was determined by HPLC (Biorad, Richmond, CA, USA). Serum creatinine, urine creatinine, serum total cholesterol, and triglyceride levels were measured using an automatic biochemistry analyser (Olympus-2000, Tokyo, Japan). Creatinine clearance was calculated. The kidneys were removed, weighed, and parts of them frozen at −80°C until processing for Western blot and RNA extraction, while other parts were removed for histological examination.

### 2.6. Optical Microscope

Kidney tissues were fixed in 10% (vol/vol) buffered formalin and embedded in paraffin. Sections (4 *μ*m) were stained with periodic acid-Schiff's reagent (PAS). The ratio of the mesangial matrix area to glomerular area (M/G) was determined by quantitative Image-Pro Plus software (PAX-it; PAXcam, Villa Park, IL, USA). Briefly, 20 glomeruli were randomly selected from each section, and positive signals within the selected glomerulus were highlighted, measured, and quantified as percent positive area of the entire glomerulus [33].

### 2.7. Electron Microscopy

Kidney samples were fixed in a mixture of 4% (wt./vol.) paraformaldehyde and 0.5% (wt./vol.) glutaraldehyde in PBS, pH 7.4, and prepared as described previously [34]. Ultrathin sections were cut, placed on a nickel grid and then examined under an electron microscope (JEM100CX-*α*, Japan).

### 2.8. Quantitative Real-Time PCR Analysis

Total RNA was extracted from renal tissues using Trizol reagent (Invitrogen, Carlsbad, CA, USA) and treated with RNase-free DNase (Invitrogen, Carlsbad, CA, USA). First-strand complementary DNA (cDNA) was generated by reverse transcriptase, with random primers (TaKaRa, Otsu, Japan). To evaluate the mRNA expression of ICAM-1, MCP-1, NF-*κ*Bp65, and TGF-*β*1 in the kidney, real-time PCR was performed using a SYBR Green master mix kit and the StepOnePlus Sequence Detection System (Applied Biosystems, Foster City, CA, USA) as previously described [35]. The sequences of the primers are described in Table 2. The 2^−ΔΔCt^ method was used to determine relative amounts of product, and data are presented as fold change, using *β*-actin as an endogenous control. 

### 2.9. Western Blot Analysis

Kidney tissue was homogenized in radioimmunoprecipitation assay buffer containing 0.5% Nonidet P-40, 0.5% sodium deoxycholate, 0.1% SDS, 10 mmol/L EDTA, and protease inhibitors. Proteins were separated by SDS-PAGE and electrotransferred to nitrocellulose membrane (Amersham, Little Chalfont, UK). After blocking in 5% nonfat milk for 1 h, membranes were incubated overnight at 4°C with primary antibody. After washing, the membrane was incubated for 1 h at room temperature with horseradish-coupled secondary antibody. The membrane-bound antibody was detected by incubation with chemiluminescent reagent plus (Perkin Elmer Life Sciences, Boston, MA, USA) and the signal captured on X-ray film. Semiquantitative analysis software (FluorChem E; ProteinSimple, CA, USA) was used to evaluate the signal.

### 2.10. Enzyme-Linked Immunosorbent Assay Analysis (ELISA)

Tumor necrosis factor *α* (TNF-*α*), IL-6 and AGEs protein levels in renal tissue were measured using commercial ELISA kits (R&D, Minneapolis, MN, USA) according to the manufacturer's instructions. 

### 2.11. Statistical Analysis

Results were expressed as mean ± SD. ANOVA was performed to compare multiple groups. When the ANOVA gave a statistically significant difference, Dunnett's test was applied; *P* < 0.05 was considered significant, and *P* < 0.01 was considered highly significant.

### 2.12. PK/PD Analysis

*X* is the matrix of the PK parameter (*P*, i.e., AUC or *C*_max⁡_) of multiple ingredients and *y* is the matrix of every effective indicator *E* (see formula (1)), where there are 1, 2, 3,…,*n* animals and parameter *P* (AUC or *C*_max⁡_) of 1, 2, 3,…,*m* ingredients:

(1) $$\begin{array}{c} X=\left[\begin{array}{cccc} P_{11} & P_{12} & \cdots & P_{1m}\\ P_{21} & P_{22} & \cdots & P_{2m}\\ \cdots & \cdots & \cdots & \cdots \\ P_{n1} & P_{n2} & \cdots & P_{nm} \end{array}\right],\;\;y=\left[\begin{array}{c} E_{1}\\ E_{2}\\ \cdots \\ E_{n} \end{array}\right]. \end{array}$$

PLS regression model between every effective indicator and the pharmacokinetic parameter of multiple ingredients can be expressed as follows:

(2) $$\begin{array}{c} y=X\times b_{\text{PLS}}+A, \end{array}$$

where *y* is every effective indicator matrix, *A* is a residual matrix, *b*_PLS_ is a regression coefficient matrix, and *X* is the matrix of the PK parameter of multiple ingredients. The best regression equation was determined by optimizing the cross-validated correlation coefficient (*Q* ^2^) using the automatic leave-one-out method to avoid overfitting the data [26].  *Q* ^2^ was calculated as follows:

(3) $$\begin{array}{c} Q^{2}=1-\frac{\sum_{i=1}^{n}{\left(y_{\text{calc}}-y_{\text{obs}}\right)}^{2}}{\sum_{i=1}^{n}{\left(y_{\text{obs}}-y_{\text{mean}}\right)}^{2}}, \end{array}$$

where *y*_calc_ is the calculated dependent variable, *y*_obs_ is the observed dependent variable, and *y*_mean_ is the mean of the observed dependent variable. PLS modelling was performed using Simca-p13 software (Umetrics AB, Umea, Sweden).

In PLS regression, the square of correlation coefficients (*R* ^2^), *Q* ^2^, ANOVA and the diagnostic plot showing calculated versus observed values of every effective indicator were used for evaluation reliability of PK/PD analysis.

Since the effects used in PK/PD analysis were all inhibitory in this study, ingredients with a negative PK parameter regression coefficient contributed to the effective indicator, whilst ingredients with positive PK regression coefficients showed no contribution to the effective indicator. Therefore, the regression coefficients of ingredient PK parameters were used to assess the relative contributions of each ingredient to every effective indicator.

The relationships between the PK parameters (AUC and *C*_max⁡_) of 8 ingredients (berberine, jatrorrhizine, palmatine, baicalin, wogonoside, wogonin, rhein, and emodin) and every quantitative effective indicator with dose-dependence were analysed using PLS regression. Either AUC or *C*_max⁡_ (the parameter with the larger *R* ^2^ and *Q* ^2^ values) was selected to explain the relationship between the PK parameters of 8 ingredients and the effective indicator.

## 3. Results

### 3.1. Pharmacokinetics of Effective XXD Ingredients in Diabetic Rats

After ig administration of XXD for 12 weeks, 11 ingredients (coptisine, jatrorrhizine, berberine, palmatine, baicalin, baicalein, wogonoside, wogonin, rhein, emodin, and aloeemodin) were determined in diabetic rat plasma. The absorption of the most active components was relatively rapid, with peak concentrations occurring at 10 min for rhein, baicalin, wogonoside, and wogonin, and at 30 min for emodin. The concentrations of coptisine, baicalein, and aloeemodin were slightly higher than the lower limit of quantification (LLOQ) at about 0.5–2 h and below the LLOQ at other times. The concentration-time curves of berberine, jatrorrhizine, palmatine, baicalin, and wogonoside exhibited double peaks in the plasma concentrations. The main PK parameters of 8 ingredients (rhein, emodin, baicalin, wogonoside, wogonin, berberine, palmatine, and jatrorrhizine) from XXD are shown in Table 3. The PK parameters of coptisine, baicalein, and aloeemodin could not be calculated because there were too few time points with detectable concentrations.

### 3.2. Effect of XXD on Metabolic Parameters in Diabetic Rats

After 12 weeks of diabetes, the levels of FBG and GAUC, HbA_1c_, serum total cholesterol, and triglyceride were significantly higher in DM than in the NC group. Compared with DM, treatment with XXD at high dose markedly lowered the FBG and serum cholesterol (Table 4). In addition, treatments with XXD at both doses significantly decreased the HbA_1c_ and serum triglyceride and improved glucose tolerance (Table 4). Similarly, significantly decreased levels of FBG, GAUC, and HbA_1c_ were also noted in animals treated with metformin, but not in those treated with losartan (Table 4).

### 3.3. Effect of XXD on Urinary Albumin Excretion and Renal Function in Diabetic Rats

**Figure 2:**
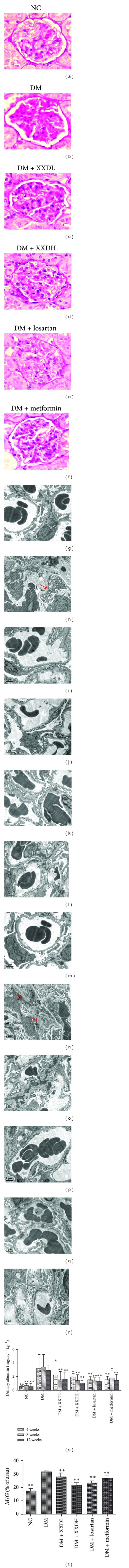
Renal pathology and urinary albumin excretion from diabetic rats treated with Xiexin decoction. (a)–(f) Periodic acid-Schiff's reagent (PAS) stain. Original magnification (a)–(f) × 400. (g)–(r) Electron microscopy (EM) analysis. Representative images of glomerular basement membrane thickening (g)–(l) and mesangial matrix expansion (m)–(r), scale bars 2 *μ*m, original magnification electron microscopy × 4,700. (s) Urinary albumin excretion at 4, 8, and 12 weeks. (t) Ratio of mesangial matrix area to glomerular area (M/G) in PAS staining. NC: normal control; DM: diabetic model control; XXDL: XXD extract 1.25 g/kg; XXDH: XXD extract 2.5 g/kg; losartan 10 mg/kg and metformin 100 mg/kg. NC and DM were treated with normal saline. All the rats were administered intragastric gavage once a day for 12 weeks. Data are expressed as mean ± SD. *P < 0.05, **P < 0.01 as compared with DM.

UAE was significantly increased at 4, 8, and 12 weeks, and creatinine clearance and the kidney weight to body weight ratio were also markedly increased at 12 weeks in the DM group, as compared with the NC group. In contrast, XXD and or losartan treatments significantly reduced UAE (Figure 2(s)), creatinine clearance, and kidney weight to body weight ratio, as compared with the DM group (Table 4). In addition, the diabetic rats treated with metformin for 12 weeks also exhibited a significant reduction in UAE (Figure 2(s)) and creatinine clearance (Table 4).

### 3.4. Effect of XXD on Renal Histopathology and Ultra-structural Pathology in Diabetic Rats

After 12 weeks of diabetes, light microscopy revealed glomerular hypertrophy, mesangial matrix expansion, and an increased M/G, as compared with the NC group, in PAS-stained kidney sections (Figures 2(a)–2(f), and 2(t)). In addition, electron microscopy of glomerular ultrastructure also revealed glomerular basement membrane thickening (Figure 2(h)) and mesangial expansion, mesangial matrix deposition (Figure 2(n)) in the DM group. However, compared with DM group, these changes were ameliorated in XXD, losartan and metformin groups (Figure 2).

### 3.5. Effect of XXD on AGEs and RAGE Expression in Diabetic Rat Kidneys

**Figure 3:**
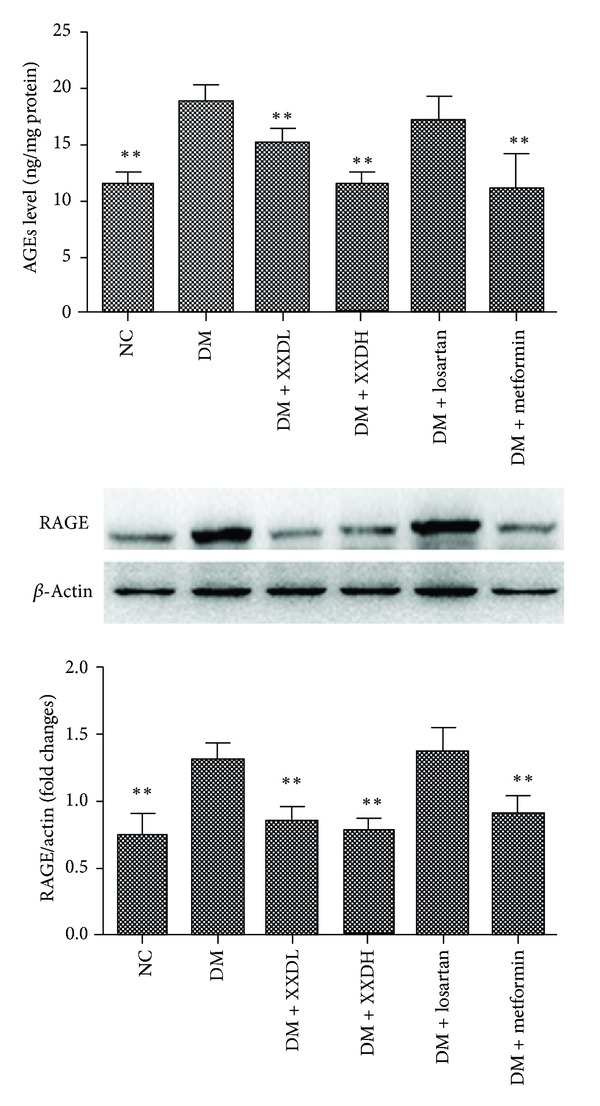
Levels of renal advanced glycation end-products (AGEs) and receptor for AGEs (RAGE) expression in diabetic rats treated with Xiexin decoction. NC: normal control; DM: diabetic model control; XXDL: XXD extract 1.25 g/kg; XXDH: XXD extract 2.5 g/kg; losartan 10 mg/kg and metformin 100 mg/kg. NC and DM were treated with normal saline. All the rats were administered via intragastric gavage once time each day for 12 weeks. Data are presented as means ± S.D, *P < 0.05, **P < 0.01 as compared with DM.

Kidney levels of AGEs and protein expression of RAGE increased in DM rats, compared with the NC group. However, treatment with XXD or metformin significantly reduced the total renal AGEs content and downregulated RAGE expression (Figure 3).

### 3.6. Effect of XXD on Renal Inflammation Factor and TGF-*β*1 Expression in Diabetic Rats

**Figure 4:**
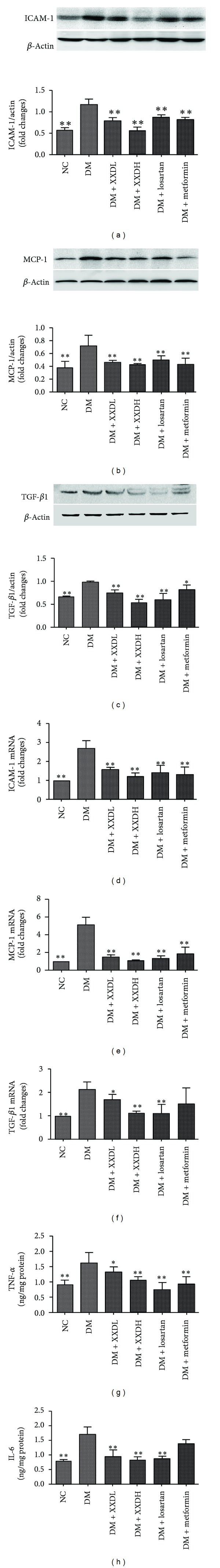
Effects of Xiexin decoction on renal inflammation factor and transforming growth factor *β*1 (TGF-*β*1) expression in diabetic rats. (a)–(c) Western blot analysis of protein levels; (d)–(f) Real-time PCR analysis of mRNA levels; (g)–(h) Quantification by ELISA. NC: normal control; DM: diabetic model control; XXDL: XXD extract 1.25 g/kg; XXDH: XXD extract 2.5 g/kg; losartan 10 mg/kg and metformin 100 mg/kg. NC and DM were treated with normal saline. All the rats were administered via intragastric gavage once time each day for 12 weeks. ICAM-1: intercellular adhesion molecule-1; MCP-1: monocyte chemotactic protein-1; TNF-*α*: tumor necrosis factor-*α*; IL-6: interleukin-6. Data are expressed as mean ± SD *P < 0.05, **P < 0.01 as compared with DM.

After 12 weeks of diabetes, renal protein and mRNA MCP-1 and ICAM-1 expression, and levels of TNF-*α* and IL-6, were markedly increased in the DM group, as compared with NC rats. Renal TGF-*β*1 protein and mRNA expression were also significantly increased in DM. XXD and losartan treatment significantly downregulated these changes (Figure 4). In addition, the diabetic rats treated with metformin also exhibited a significant reduction in MCP-1 and ICAM-1 expression and TNF-*α* level (Figure 4). Collectively, these data indicated that XXD could suppress the renal inflammation induced by diabetes. 

### 3.7. Effect of XXD on Renal NF-*κ*B Signaling Pathway in Diabetic Rats

**Figure 5:**
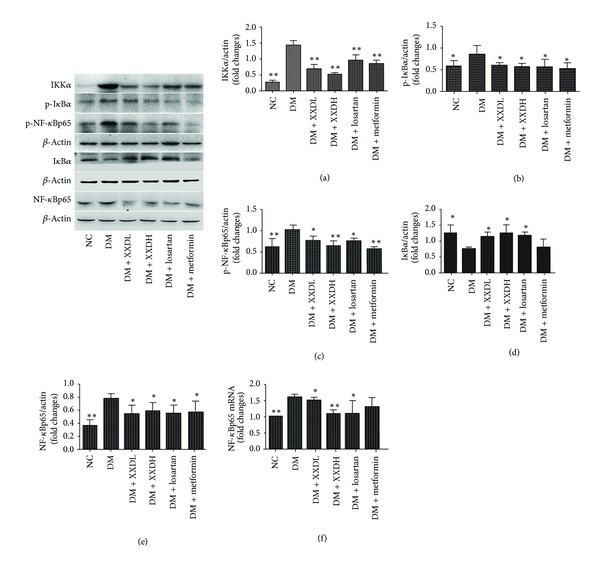
Effect of Xiexin decoction on renal nuclear factor-*κ*B (NF-*κ*B) signalling pathway in diabetic rats. (a)–(e) Western blot analysis of protein levels. (g) Real-time PCR analysis of mRNA levels. NC: normal control; DM: diabetic model control; XXDL: XXD extract 1.25 g/kg; XXDH: XXD extract 2.5 g/kg; Losartan 10 mg/kg and metformin 100 mg/kg. NC and DM were treated with normal saline. All the rats were administered via intragastric gavage one time each day for 12 weeks. IKK*α*: inhibitor of nuclear factor-*κ*B kinase subunit *α*; I*κ*B*α*, inhibitor of nuclear factor-*κ*B subunit *α*; p-I*κ*B*α*: phospho-I*κ*B*α*; NF-*κ*Bp65: nuclear factor-*κ*Bp65; p-NF-*κ*Bp65: phospho-NF-*κ*Bp65. Data are expressed as mean ± SD, *P < 0.05, **P < 0.01 as compared with DM.

After 12 weeks of diabetes, increased protein expression of renal IKK*α*, phospho-I*κ*B*α*, phospho-NF-*κ*Bp65, and NF-*κ*Bp65, with decreased I*κ*B*α* expression, was observed in DM rats, compared with the NC group. XXD and losartan treatments significantly ameliorated these changes. In addition, the increased renal NF-*κ*Bp65 mRNA expression in diabetic rats was downregulated by XXD and losartan treatment (Figure 5). These findings suggested that XXD treatment could suppress activation of the renal NF-*κ*B signalling pathway in diabetic rats.

### 3.8. PK/PD Relationships

**Figure 6:**
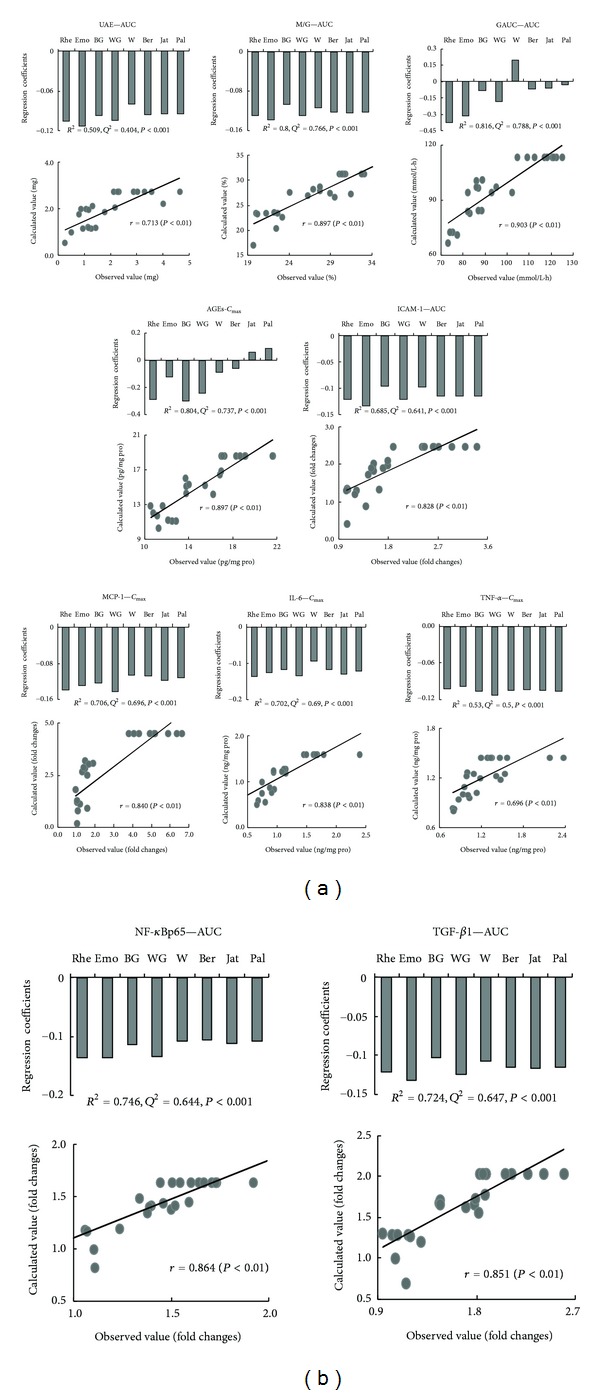
Reliability evaluation for the pharmacokinetics/pharmacodynamics analysis using partial least squares models and regression coefficients between pharmacokinetic parameter of ingredients with effective indicators in diabetic nephropathy rats treated with XXD for 12 weeks. *R* ^2^: square of correlation coefficients; *Q* ^2^: the cross-validated correlation coefficients, *P*: significance using ANOVA. Rhe: Rhein; Emo: emodin; BG: baicalin; WG: wogonoside; W: wogonin; Ber: berberine; Jat: jatrorrhizine; Pal: palmatine. *C*_max⁡_: the maximum plasma concentration; AUC: the area under the curve of plasma concentration of ingredient; UAE: Urinary albumin excretion; M/G: ratio of mesangial matrix area to glomerular area; GAUC: area under the blood glucose response curve; AGEs: advanced glycation end-products; ICAM-1: intercellular adhesion molecule-1; MCP-1: monocyte chemotactic protein-1; IL-6: interleukin-6; TNF-*α*: tumor necrosis factor-*α*; NF-*κ*Bp65: nuclear factor-*κ*Bp65; TGF-*β*1: transforming growth factor *β*1. ICAM-1, MCP-1, TGF-1, and NF-*κ*B panels show mRNA expression.

Using PLS models analysis, *R* ^2^, *Q* ^2^, ANOVA *P* values, and a diagnostic plot showing the calculated effect values from the PK parameters of 8 ingredients, versus the observed effect values for each of 10 quantitative effective indicators, are summarized in Figure 6. The relationships all appeared to show reasonable correlations (*R* ^2^ range 0.509–0.816), evaluation performances (*Q* ^2^ range 0.404–0.788), and significant ANOVA (*P* < 0.01). The differences between the *R* ^2^ and *Q* ^2^ values (*<*0.11) were moderate, indicating sufficient model reliability. Good agreement for all models was observed. From the regression coefficients of PK parameters of 8 ingredients (Figure 6), we found that 8 XXD constituents (berberine, jatrorrhizine, palmatine, baicalin, wogonoside, wogonin, rhein and emodin) made significant contributions to the renal protection (reduced UAE, M/G, renal TNF-*α*, and IL-6 level and inhibited MCP-1, ICAM-1, TGF-*β*1 and NF-*κ*B p65 expression) observed in diabetic rats. Seven constituents (berberine, jatrorrhizine, palmatine, baicalin, wogonoside, rhein, and emodin) were found to make significant contributions to the improvement of glucose tolerance, and 6 constituents (berberine, baicalin, wogonoside, wogonin, rhein, and emodin) made significant contributions to the decrease in renal AGEs in diabetic rat kidneys. 

## 4. Discussion

This research showed that rats where diabetes was induced by high-fat diet and streptozotocin for 12 weeks exhibited a number of characteristics of early DN, including glucolipid metabolism disorder, increased UAE, high glomerular filtration, glomerular mesangial matrix proliferation, and basement membrane thickening. XXD exhibited an anti-early DN effect, as it improved the above changes. 

**Figure 7:**
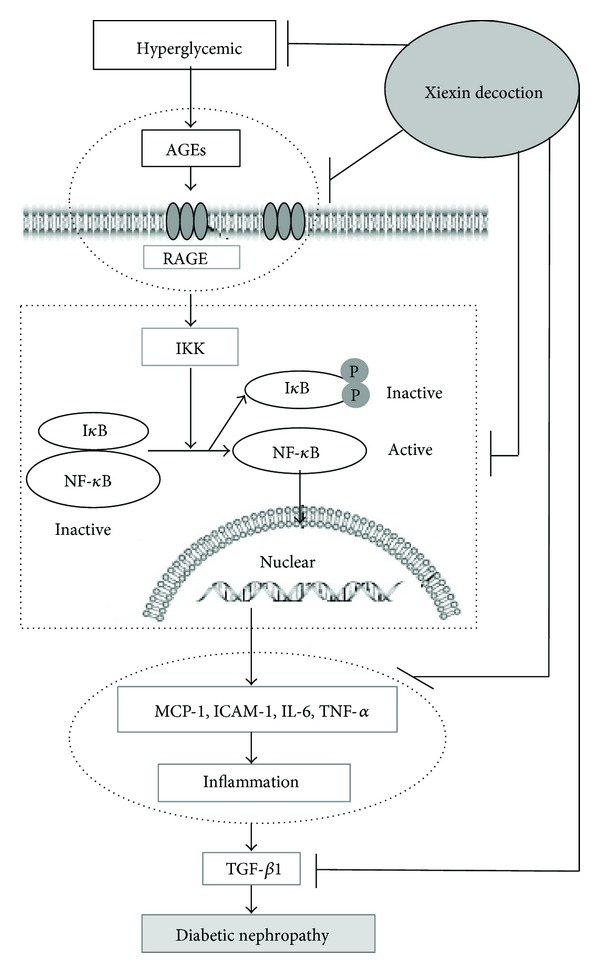
Proposed molecular mechanisms underlying the renal protective role of Xiexin decoction in diabetic nephropathy rats. AGEs: advanced glycation end-products; RAGE: receptor for AGEs; IKK: inhibitor of nuclear factor-*κ*B kinase; I*κ*B: inhibitor of nuclear factor-*κ*B; NF-*κ*B: nuclear factor-*κ*B; P: phosphorylation; ICAM-1: intercellular adhesion molecule-1; MCP-1: monocyte chemotactic protein-1; IL-6: interleukin-6; TNF-*α*: tumor necrosis factor-*α*; TGF-*β*1: transforming growth factor *β*1. *⊢*: inhibition.

Our data indicated that in diabetic rat kidneys, renal AGEs and RAGE increased. This would be predicted to activate the downstream I*κ*B kinase, promoting I*κ*B phosphorylation and I*κ*B degradation and allowing NF-*κ*Bp65 to be released and phosphorylated. The phosphorylated NF-*κ*Bp65 would upregulate target gene expression, such as inflammatory cytokines and cell adhesion molecules, including IL-6, TNF-*α*, MCP-1, and ICAM-1. The resulting increase in kidney inflammation could further promote renal TGF-*β*1 expression, which enhanced the accumulation of glomerular mesangial extracellular matrix and mesangial expansion, resulting in the development of DN (Figure 7). These results were similar to the pathogenesis of DN reported in the literature [6, 8], whereby the long-term hyperglycaemia found in the diabetic state could induce AGEs accumulation in the kidney, activating RAGE and subsequently the NF-*κ*B inflammatory pathway. Moreover, the resulting kidney inflammation can promote DN progression [8, 33]. The results of the present study, therefore, indicated that the molecular mechanism underlying XXD's anti-DN activity related to its ability to decrease renal AGEs, downregulate RAGE expression, inhibit NF-*κ*B pathway activation, inflammatory factor formation, and TGF-*β*1 expression, thus preventing kidney injury (Figure 7). 

Because DN is a complicated disease, it has proved difficult to treat using a single compound acting on a single target. The present study found, through combined PK/PD analysis of the relationships between the PK parameters of XXD ingredients and their anti-DN effects, that multiple active ingredients of XXD acted on multiple targets *in vivo* to produce an overall comprehensive anti-DN effect. In recent years, network pharmacology and multipharmacology research studies have shown that multiple active ingredients in TCM may act on multiple targets within the diabetic network to generate an overall comprehensive effect [36]. However, *in vivo* studies are essential to determine whether the active ingredients predicted by computer are correct because their pharmacological properties can be affected by the concentrations achieved *in vivo* and interactions between ingredients. This study provided an appropriate research method for analysis of the active ingredients in TCM and their mechanisms of action, through combined PK/PD analysis using PLS regression *in vivo*.

Several studies have reported that rhein [37], emodin [38], baicalin [39], and berberine [40, 41] exhibited anti-DN effects. Rhein and baicalin could downregulate renal TGF-*β*1 protein expression [37, 39]. Berberine increased IKB*α* and decreased NF-*κ*Bp65 protein levels in diabetic mouse kidney, as well as inhibiting renal AGE generation and downregulating TGF-*β*1, ICAM-1, and VCAM-1 protein expression [40, 41]. However, the mechanisms of action of these TCM ingredients and the concentrations achieved *in vivo* were unclear prior to the present study. In addition, our results showed that wogonoside, wogonin, palmatine, and jatrorrhizine also exhibited anti-DN activity and illustrated their mechanisms of action, indicating that these ingredients are worthy of further study. 

In the present PLS analysis, we found that the *C*_max⁡_ or AUC of 8 ingredients had a poor correlation with the observed effect indicators, which lacked dose dependency, such as FBG, HbA_1c_, serum triglyceride, serum cholesterol, creatinine clearance and kidney weight/body weight. Among these indicators, reduced blood lipid levels may relate to local effects of XXD ingredients on the gut, because it was reported that *Rhizoma coptidis* and berberine reduced blood lipid levels by regulation of gut microbes [42, 43]. The other reasons call for further studies. This phenomenon also showed that alternative approaches need to be developed for PK/PD analysis of effect indicators without dose dependency.

In the present study, we used metformin and losartan as two positive control drugs to evaluate the reliability of the DN model. Metformin has a hypoglycaemic effect and losartan has a renal protective effect. Our data indicated that both exhibited an anti-DN effect, losartan via inhibition of NF-*κ*B signalling activity and reduction in levels of inflammatory factors, and metformin via improving the glucolipid metabolism disorder, decreasing AGEs, and suppressing expression of RAGE and inflammatory molecules. These results are in agreement with those of previous studies [44–48].

## 5. Conclusion

In conclusion, XXD exhibited an anti-DN effect via inhibition of renal inflammation, mediated via NF-*κ*B signalling as well as inhibition of renal AGEs accumulation and expression of its receptor. Based on the combined PK/PD analysis using PLS regression, XXD was found to act on multiple targets to generate an overall anti-DN effect. This study provides a foundation for further research and development of XXD. Furthermore, this study demonstrated an effective experimental approach to analysis of the active ingredients in herbal compounds.

## Figures and Tables

**Table 1:** Contents of the Xiexin decoction ingredients.

Ingredients	Content (mg/g)
Baicalin	32.1 ± 1.3
Berberine	7.2 ± 0.5
Wogonoside	5.8 ± 0.5
Baicalein	3.3 ± 0.1
Coptisine	2.4 ± 0.2
Palmatine	2.2 ± 0.1
Jatrorrhizine	1.8 ± 0.2
Wogonin	1.8 ± 0.3
Rhein	0.26 ± 0.04
Emodin	0.029 ± 0.002
Aloeemodin	0.028 ± 0.003

**Table 2:** Nucleotide sequence of primers used in real-time PCR.

Gene	Primers	Nucleotide sequence 5′-3′
ICAM-1	Forward; reverse	AGGTATCCATCCATCCCAC; GCCGAGGTTCTCGTCTTC
MCP-1	Forward; reverse	TCTCTTCCTCCACCACTATGCA; GGCTGAGACAGCACGTGGAT
TGF-*β*1	Forward; reverse	GCTAATGGTGGACCGCAACAAC; TCTGGCACTGCTTCCCGAATG
NF-*κ*Bp65	Forward; reverse	GGCAGCACTCCTTATCAA; GGTGTCGTCCCATCGTAG
*β*-actin	Forward; reverse	TTATCGGCAATGAGCGGTTC; AGCACTGTGTTGGCATAGAG

ICAM-1: intercellular adhesion molecule-1; MCP-1: monocyte chemotactic protein-1; TGF-*β*1: transforming growth factor-*β*1; NF-*κ*Bp65: nuclear factor *κ*Bp65.

**Table 3:** Pharmacokinetic parameters of effective ingredients after intragastric gavage of Xiexin decoction for 12 weeks in diabetic rats.

Constituents	1.25 g/kg		2.5 g/kg	
	*C*_max⁡_ (*μ*g/mL)	AUC_0–24 h_ (*μ*g·h/mL)	*C*_max⁡_ (*μ*g/mL)	AUC_0–24 h_ (*μ*g·h/mL)
Rhein	0.961 ± 0.430	1.102 ± 0.216	1.753 ± 0.514	2.789 ± 0.937
Emodin	0.013 ± 0.007	0.061 ± 0.008	0.022 ± 0.009	0.119 ± 0.025
Baicalin	1.564 ± 0.570	4.696 ± 1.734	4.309 ± 1.709	16.067 ± 9.597
Wogonoside	1.137 ± 0.321	3.458 ± 0.894	2.240 ± 0.475	8.884 ± 2.992
Wogonin	0.047 ± 0.038	0.154 ± 0.071	0.149 ± 0.086	0.423 ± 0.307
Berberine	0.051 ± 0.020	0.757 ± 0.389	0.118 ± 0.078	1.727 ± 1.037
Palmatine	0.017 ± 0.013	0.113 ± 0.069	0.029 ± 0.018	0.312 ± 0.197
Jatrorrhizine	0.014 ± 0.009	0.172 ± 0.057	0.021 ± 0.013	0.201 ± 0.152

Data are expressed as mean ± SD, *n* = 8 in each group.

**Table 4:** Effect of Xiexin decoction on metabolic parameters and renal function in diabetic rats.

Groups	NC	DM	DM + XXDL	DM + XXDH	DM + losartan	DM + metformin
FBG (mmol/L)	3.0 ± 0.5**	22.3 ± 2.8	21.0 ± 3.3	17.2 ± 2.9*	21.6 ± 4.4	9.4 ± 2.5**
HbA_1c_ (%)	4.0 ± 0.1**	7.2 ± 0.3	6.6 ± 0.9*	6.6 ± 0.2*	6.9 ± 0.2	6.6 ± 0.5**
GAUC (mmol/L × h)	28.6 ± 4.7**	117.1 ± 6.4	89.9 ± 6.4**	77.4 ± 10.7**	111.8 ± 11.1	70.9 ± 9.5**
Serum triglyceride (mmol/L)	0.6 ± 0.2**	1.1 ± 0.6	0.6 ± 0.2*	0.5 ± 0.1**	0.7 ± 0.3	1.0 ± 0.3
Serum cholesterol (mmol/L)	1.1 ± 0.2**	1.5 ± 0.2	1.2 ± 0.2	1.1 ± 0.2**	1.3 ± 0.2	1.2 ± 0.4
Creatinine clearance (mL min^−1^kg^−1^)	3.1 ± 1.5**	7.5 ± 3.9	3.6 ± 0.9**	3.7 ± 1.9**	4.8 ± 3.6*	4.6 ± 2.7*
Kidney weight/body weight (%)	0.58 ± 0.05**	1.18 ± 0.06	1.12 ± 0.16	1.04 ± 0.10*	1.00 ± 0.05*	1.10 ± 0.04

NC: normal control; DM: diabetic model control; XXDL: XXD extract 1.25 g/kg; XXDH: XXD extract 2.5 g/kg; losartan 10 mg/kg and metformin 100 mg/kg. NC and DM were treated with normal saline. All the rats were administered via intragastric gavage once time each day for 12 weeks. FBG: fasting blood glucose; GAUC: area under the blood glucose response curve. Data are expressed as mean ± SD; *n* = 8–10. *P < 0.05 and **P < 0.01 as compared with DM.

## Abbreviations

AGE: Advanced glycation end-product
*C*_max⁡_: The maximum plasma concentration
DN: Diabetic nephropathy
FBG: Fasting blood glucose
GAUC: Area under the blood glucose response curve
ICAM-1: Intercellular adhesion molecule-1
I*κ*B*α*: Inhibitor of nuclear factor *κ*B subunit *α*
IKK*α*: Inhibitor of nuclear factor *κ*B kinase subunit *α*
LC/MS/MS: Liquid chromatography and tandem mass spectrometry
MCP-1: Monocyte chemotactic protein-1
M/G: Ratio of the mesangial matrix area to glomerular area
NF-*κ*B: Nuclear factor *κ*B
PK/PD: Pharmacokinetic/pharmacodynamic
PLS: Partial least squares
PAS: Periodic acid-Schiff's reagent
*Q*^2^: The cross-validated correlation coefficient
RAGE: Receptor for advanced glycation end-product
*R*^2^: Square of correlation coefficients
TCM: Traditional Chinese medicine
TNF-*α*: Tumor necrosis factor *α*
TGF-*β*1: Transforming growth factor-*β*1
UAE: Urinary albumin excretion
XXD: Xiexin decoction.
